# Ancient Schwannoma Presenting at the Cauda Equina: A Report of a Rare Case

**DOI:** 10.7759/cureus.40430

**Published:** 2023-06-14

**Authors:** Hayes B Fountain, G Damian Brusko, Andrew E Rosenberg, Adam S Levy, Joacir G Cordeiro

**Affiliations:** 1 Department of Neurological Surgery, University of Miami Miller School of Medicine, Miami, USA; 2 Department of Pathology, University of Miami Miller School of Medicine, Miami, USA

**Keywords:** intradural extramedullary ancient schwannoma, extramedullary spinal mass, verocay bodies, target lesion mri, ancient schwannoma of cauda equina, antoni b, antoni a, common schwannoma, cauda equina, ancient schwannoma

## Abstract

Ancient schwannoma is an uncommon variant of schwannoma. While many reports have presented defining histologic and clinical features of ancient schwannoma, there are only a very few cases in the literature, to our knowledge, of ancient schwannoma presenting at the cauda equina. The current report of ancient schwannoma presenting at the cauda equina adds to the literature and discusses the identification of specific histologic characteristics, the role of conservative medical management, surgical resection, and prognostication in this select subset of patients.

## Introduction

Schwannomas are benign, encapsulated, slow-growing nerve sheath tumors composed of Schwann cells [1]. Ancient schwannoma is an uncommon variant of schwannoma that accounts for less than 1% of schwannoma [2]. The term “ancient" refers to histologic characteristics that suggest that the neoplasm is of long duration. Classically, the features of ancient schwannoma include those that involve stroma and neoplastic cells [2-5]. On magnetic resonance imaging (MRI), greater homogeneity of the mass is seen in ancient schwannoma compared to classic schwannoma due to the increased admixing of Antoni A and B areas [5,6]. Biologically, these tumors are relatively slow-growing, and malignant transformation is very uncommon [7]. Ancient schwannoma tends to arise in the head, neck, pelvis, retroperitoneum, and limbs and less frequently in the spinal canal [2-6,8-14]. Involvement of the cauda equina is extremely rare. To the best of our knowledge, there are only three other reported cases of ancient schwannoma of the cauda equina [2,8,14]. We present the fourth case of a successfully treated ancient schwannoma of the cauda equina to add to the limited literature on this exceedingly rare phenomenon. 

## Case presentation

**Figure 1:**
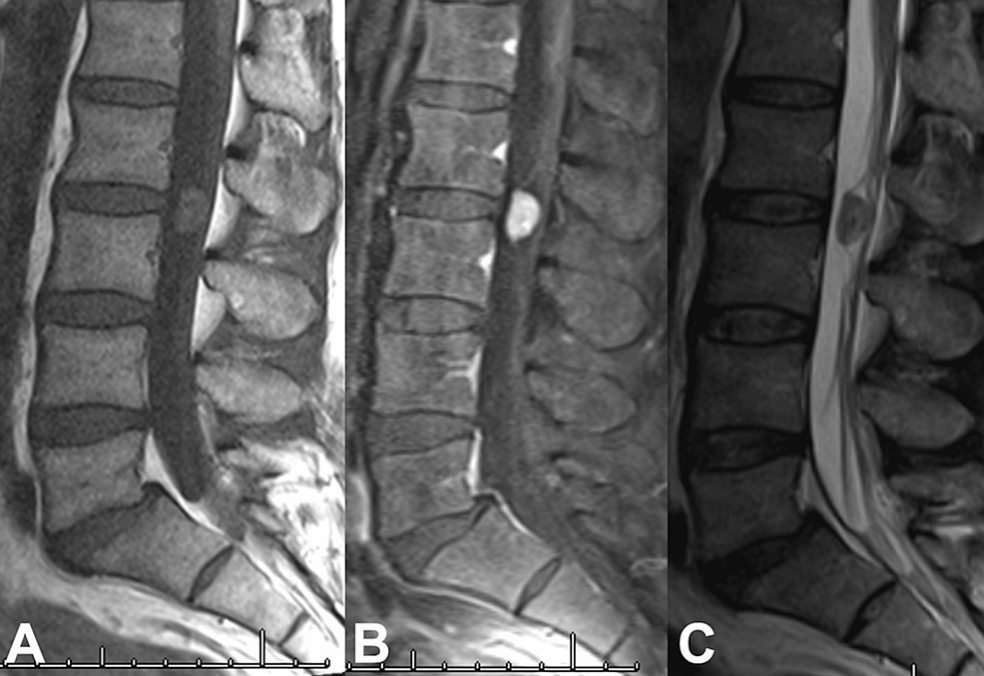
Preoperative MRI of the cauda equina showing: (A) sagittal T1 without contrast; (B) sagittal T1 with contrast; (C) sagittal T2.

**Figure 2:**
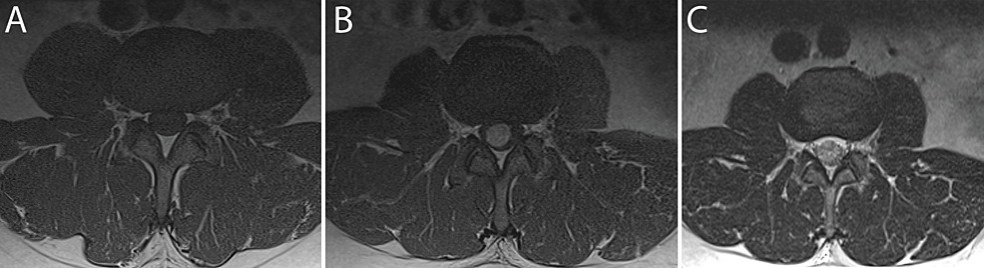
Preoperative MRI of the cauda equina showing: (A) axial T1 without contrast; (B) axial T1 with contrast; (C) axial T2.

A 51-year-old male patient with chronic axial lower back pain and recent radicular pain was referred to the neurological surgery service given imaging findings concerning for a spinal mass. He described experiencing lower back pain with right-sided sciatica refractory to conservative management. He was neurologically intact on examination. MRI demonstrated a well-circumscribed, contrast-enhancing 1.5 cm intradural, extramedullary lesion at the level of L2 compressing the cauda equina (Figures 1-2). Given the progressive symptoms congruent with the imaging findings, surgical resection was elected as the treatment of choice. He underwent an L2-L3 laminectomy guided by fluoroscopy and intraoperative ultrasound. The microscopy-assisted procedure yielded a gross total resection of the lesion. Intraoperatively, the tumor was found to occupy nearly the entire spinal canal with significant compression of the nerve roots. No complications occurred during the case. Postoperative MRI demonstrated complete resection of the lesion.

**Figure 3:**
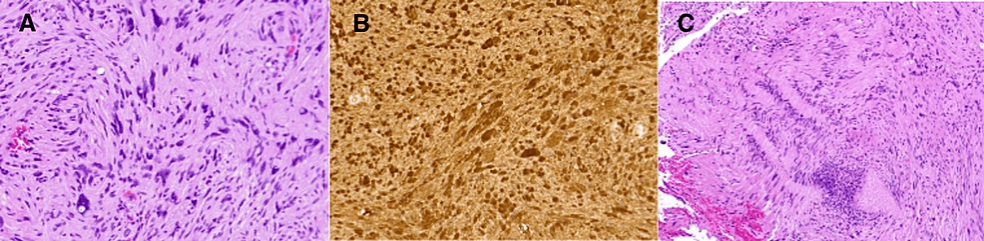
(A) Pathology of the lesion using hematoxylin and eosin staining showing areas of ancient change with nuclear pleomorphism, hyperchromasia, and intranuclear vacuolation; (B) S100 immunostaining of the lesion; (C) Hematoxylin and eosin stain showing areas of classic morphology with Verocay bodies.

Pathological evaluation revealed ancient schwannoma (Figure 3). The tumor was composed of an admixture of Antoni A and B regions with scattered tumor cells having enlarged, hyperchromatic nuclei that contained intranuclear inclusions (Figure 2A). There was no significant mitotic activity. Immunohistochemistry revealed that the tumor cells were positive for SOX10 and expression of H3k27me3 was maintained. The proliferation rate estimated by the Ki67 nuclear labeling indexes was 3%.

## Discussion

We report the occurrence of an intradural, extramedullary tumor from the cauda equina that was seen to be an ancient schwannoma. This is an uncommon pathological variant, especially in this location, and this is the fourth case of ancient schwannoma arising in the cauda equina that has been reported in the literature [2,8,14]. Our patient underwent successful resection without permanent complications and had symptomatic improvement. 

Ancient schwannoma is uncommon, making up less than 1% of all schwannomas [3]. Ancient schwannoma of the cauda equina usually presents with the symptom of lumbar pain (Table 1). Saiful Azli et al. [2] reported a 54-year-old male who also had two years of sciatic pain. Wierzbicki et al. [14] reported a 36-year-old male with one year of leg pain. In our case, the presentation was four years of chronic axial back pain in the lumbar region progressing to radicular pain. The longer duration of symptoms before imaging and subsequent resection in the current case is likely due to the typical insidious nature of the development of these lesions that can take large amounts of time before becoming significantly symptomatic. In two of the three previously reported cases, the patients were male (aged 36 and 54 years, respectively); the third case was of a 39-year-old female. Males may potentially be more predisposed to ancient schwannoma of the cauda equina, given the increased prevalence of spinal schwannomas in males [15]. These tumors are usually associated with a long history of mild lower back pain that worsens progressively over years causing neurological symptoms. Though these symptoms may vary, possible symptoms of acute compression of the nerve roots by the lesion are claudication, change in perianal sensation, and bowel or bladder disfunction [16].

**Table 1:** Cases of ancient schwannoma of the cauda equina reported in detail

Author	Age	Sex	Location	Symptoms	Duration	Therapy	Recovery
Saiful Azli et al. [2]	54	M	Conus medullaris-cauda equina	Worsening lumbar pain and sciatica	2 years	Surgical	Complete
Domínguez et al. [8]	39	F	Intrasacral, cauda equina involvement	Lumbar pain	Not reported	Surgical	Complete
Wierzbicki et al. [14]	36	M	Cauda equina	Lumbar pain and cruralgia	1 year	Surgical	Complete
Current report	51	M	Cauda equina	Lumbar pain and sciatica	3-4 years	Surgical	Complete

As for other intraspinal masses, gadolinium-enhanced MRI is standard for investigation [2,6,14]. Classically, a “target” appearing lesion will be seen for classic schwannoma on MRI with a hyperintense exterior capsule and hypointense internal core [6,10]. This unique radiologic finding is important in distinguishing ancient schwannoma from classic schwannoma, as classic schwannoma are more heterogenous radiologically [6,10]. The heterogeneity seen in classic schwannoma is due to the two different types of substrates and their disproportional composition in the exterior capsule and internal core of the lesion [6]. The exterior capsule will be hypointense on T1 weighted images (T1w) and hyperintense on T2 weighted images (T2w) indicating increased prevalence of Antoni B areas. Conversely, the internal core of classic schwannoma will generally be isointense or hypointense on both T1w and T2w, with a strong gadolinium enhancement indicating increased prevalence of Antoni A areas [6]. These two characteristic areas make up the “target” appearance that is present in greater than 50% of benign peripheral nerve sheath tumors [10]. In ancient schwannoma, the Antoni A and B areas are more finely admixed, as seen in our case with a relatively homogenous-appearing lesion on MRI imaging in both T1w and T2w. This homogeneity in ancient schwannoma imaging makes it difficult to differentiate them from other tumors that may have similar features [6,10]. Histological evaluation is the gold standard for diagnosis. Imaging follow-up can be performed in mild oligosymptomatic stable lesions. 

Both classic and ancient schwannomas are well circumscribed, have Antoni A and B regions, and have limited mitotic activity. The differences in histology of these tumors is that there is greater intermixing of the Antoni A and B regions in ancient schwannoma and the stroma shows more hyalinization, cystic change, calcification, and the cytological changes of nuclear megaly, hyperchromasia, and intranuclear vacuolization and inclusions [2,3,11]. 

In every reported case of ancient schwannoma of the cauda equina, the treatment of choice was surgical resection after the failure of conservative treatment. Conservative treatment with medical pain control and follow-up imaging is an option as long as the pain is reasonably well controlled and the patient does not present any neurological deficits. Surgical excision is the definitive treatment for ancient schwannoma of the cauda equina. In cases with frank spinal cord or cauda equina compression, early surgery is recommended [16]. However, due to the slow-growing nature of ancient schwannoma and the low likelihood of malignant transformation, observation may be an acceptable alternative in cases where there are no prominent neurological deficits and spinal cord compression is absent on MRI imaging. There is no role for chemotherapy or radiation therapy. 

## Conclusions

We described the case of an adult male with a symptomatic lesion originating from the cauda equina and presenting with mass effect. Surgical resection was performed and the final pathology showed ancient schwannoma of the cauda equina. Our case highlights the importance of including ancient schwannoma in the differential diagnosis in patients with intradural, extramedullary spinal lesions with a homogeneous appearance on MRI imaging. Meticulous microsurgical resection coupled with pathological analysis is paramount for optimal patient care.

## References

[1] Joshi R (2012). Learning from eponyms: Jose Verocay and Verocay bodies, Antoni A and B areas, Nils Antoni and schwannomas. Indian Dermatol Online J.

[2] Saiful Azli MN, Abd Rahman IG, Md Salzihan MS (2007). Ancient schwannoma of the conus medullaris. Med J Malaysia.

[3] Darwish BS, Balakrishnan V, Maitra R (2002). Intramedullary ancient schwannoma of the cervical spinal cord: case report and review of literature. J Clin Neurosci.

[4] Harazono Y, Kayamori K, Sakamoto J (2022). Retrospective analysis of schwannoma in the oral and maxillofacial region: clinicopathological characteristics and specific pathology of ancient change. Br J Oral Maxillofac Surg.

[5] Hide IG, Baudouin CJ, Murray SA, Malcolm AJ (2000). Giant ancient schwannoma of the pelvis. Skeletal Radiol.

[6] Lee YS, Kim JO, Park SE (2010). Ancient schwannoma of the thigh mimicking a malignant tumour: a report of two cases, with emphasis on MRI findings. Br J Radiol.

[7] Shimada Y, Miyakoshi N, Kasukawa Y, Hongo M, Ando S, Itoi E (2006). Clinical features of cauda equina tumors requiring surgical treatment. Tohoku J Exp Med.

[8] Domínguez J, Lobato RD, Ramos A, Rivas JJ, Gómez PA, Castro S (1997). Giant intrasacral schwannomas: report of six cases. Acta Neurochir (Wien).

[9] Ishihara T, Ono T (2001). Degenerated neurilemoma (ancient schwannoma). J Dermatol.

[10] Isobe K, Shimizu T, Akahane T, Kato H (2004). Imaging of ancient schwannoma. AJR Am J Roentgenol.

[11] Hsieh CT, Tsai WC, Liu MY (2011). Intradural lumbar cystic schwannoma. Neurosciences (Riyadh).

[12] Hayashi F, Sakai T, Sairyo K, Hirohashi N, Higashino K, Katoh S, Yasui N (2009). Intramedullary schwannoma with calcification of the epiconus. Spine J.

[13] Ross DA, Edwards MS, Wilson CB (1986). Intramedullary neurilemomas of the spinal cord: report of two cases and review of the literature. Neurosurgery.

[14] Wierzbicki V, Pesce A, Marrocco L, Piccione E, Frati A, Caruso R (2016). Ancient schwannoma of the cauda equina: our experience and review of the literature. Case Rep Surg.

[15] Tish S, Habboub G, Lang M (2019). The epidemiology of spinal schwannoma in the United States between 2006 and 2014. J Neurosurg Spine.

[16] Qureshi A, Sell P (2007). Cauda equina syndrome treated by surgical decompression: the influence of timing on surgical outcome. Eur Spine J.

